# Samsum ant venom modulates the immune response and redox status at the acute toxic dose *in vivo*

**DOI:** 10.1590/1678-9199-JVATITD-2019-0020

**Published:** 2019-12-02

**Authors:** Hossam Ebaid, Bahaa Abdel-Salam, Ibrahim Alhazza, Jameel Al-Tamimi, Iftekhar Hassan, Ahmed Rady, Ashraf Mashaly, Ahmed Mahmoud, Reda Sammour

**Affiliations:** 1 Department of Zoology, College of Science, King Saud University, Riyadh 11451, Saudi Arabia.; 2 Department of Biology, College of Science and Humanities in El-Quwiaya, 11961, Shaqra University, Saudi Arabia.

**Keywords:** Samsum ant venom, Polymorphonuclear cells (PMNs), Costimulatory molecules (CD80 and CD86), Major histocompatibility complex (MHC), MHC-II, Interferon gamma (INF-γ), Interleukin-17 (IL-17)

## Abstract

**Background:**

Ant venoms express surface molecules that participate in antigen presentation involving pro- and anti-inflammatory cytokines. This work aims to investigate the expression of MHC-II, CD80 and CD86 on the polymorphonuclear cells (PMNs) in rats injected with samsum ant venom (SAV).

**Methods:**

Rats were divided into three groups - control, SAV-treated (intraperitoneal route, 600 μg/kg), and SAV-treated (subcutaneous route, 600 μg/kg). After five doses, animals were euthanized and samples collected for analysis.

**Results:**

The subcutaneous SAV-trated rats presented decreased levels of glutathione with increased cholesterol and triglyceride levels. Intraperitoneal SAV-treated animals displayed significantly reduced concentrations of both IFN-γ and IL-17 in comparison with the control group. However, intraperitoneal and subcutaneous SAV-treated rats were able to upregulate the expressions of MHC-II, CD80 and CD86 on PMNs in comparison with the control respectively. The histological examination showed severe lymphocyte depletion in the splenic white pulp of the intraperitoneal SAV-injected rats.

**Conclusion:**

Stimulation of PMNs by SAV leads to upregulation of MHC-II, CD 80, and CD 86, which plays critical roles in antigen presentation and consequently proliferation of T-cells. Subcutaneous route was more efficient than intraperitoneal by elevating MHC-II, CD80 and CD86 expression, disturbing oxidative stability and increasing lipogram concentration.

## Background

Polymorphonuclear cells (PMNs) possess a short half-life in the circulatory system because they constitutively undergo apoptosis [1]. Under certain conditions, PMNs play a vital role in the effector arm of host immune defense through clearance of immune complexes, phagocytosis of opsonized particles, and release of inflammatory mediators [2]. Traditionally, considered to be the first line of defense against bacterial infection, it is clear that PMNs also participate in chronic inflammation and regulation of the immune response when appropriately activated [3]. PMN infiltration plays a pivotal role in inflammation that is also attributive of tissue damage during the inflammatory response [4]. During infection, the host immune system activates the inflammatory response by attracting the neutrophils to the site of infection. The immune cells engulf the cell debris and release ROS to heighten immune response. It further attracts pro-inflammatory macrophages (M1) that engulf the neutrophils as well as trigger enhanced production of ROS that leads to a transition from M1 to M2 (anti-inflammatory macrophages). M2 finally release many anti-inflammatory cytokines including IL-10 and tissue growth factors to promote tissue repair [5, 6]. Besides, through interactions with various cells of the immune system, such as antigen-presenting cells and lymphocytes, neutrophils, M2 also influence inflammatory responses [7, 8].

The samsum ant *Brachyponera sennaarensis* (Formicidae: Ponerinae) is primarily found in many parts of Saudi Arabia. The sting of this ant generally results in pain, inflammation, and irritation in humans. However, sometimes, it can lead to severe allergic reactions ranging from mild ones to anaphylactic shock [9, 10]. Despite its documented adverse effects, the toxin at precise doses shows promising pharmacological properties [11]. In addition, we have previously hypothesized that samsum ant venom (SAV) can induce acute toxic inflammation via activation of PMNs as part of their mechanism of toxic effects *in vivo*.

Moreover, PMNs can express MHC-II and co-stimulatory molecules (CD80 and CD86) surface molecules [12, 13]. Under certain stimulatory states, PMNs can present MHC-II-restricted antigens [14] or can acquire MHC-II antigens in the course of the disease [15]. It is likely that the change of MHC-II into PMN may serve as a novel diagnostic marker for active immune responses. Therefore, the present study was conducted to evaluate the expression of MHC-II and co-stimulatory CD80 and CD86 on PMNs in response to host exposure to the SAV to ascertain if these cells are indeed activated during SAV induced host inflammatory response. In addition, the study also investigated if SAV is more efficient by subcutaneous (SC) route or intraperitoneal (IP) injection.

## Methods

### Collection of samsum ants and dissection of the venom glands

Samsum ant colonies were collected from Hotat Bani Tamim Governorate, Kingdom of Saudi Arabia. The ants were placed in plastic containers (20 × 70 cm) with upper interior edges painted with grease to prevent their exit. A glass tube was inserted into each case to provide a 10% sugar solution twice a day. At the time of venom harvest, ants were dissected and their sting apparatus was dettached by grabbing the last segment of the abdomen and detaching it with the sting apparatus under microscopic magnification. Venom glands were pooled, homogenized and the resulting mash was then centrifuged at 1000 rpm for two minutes at 4°C [16]. The resulting supernatant was collected and lyophilized into powder. SAV dry powder was diluted in phosphate buffered saline (PBS, pH 7.4) at the concentration of 2 mg of venom in 1000 µL PBS. The venom solution was stored at -25ºC until use. On average, 0.5-2 µL of venom can be extracted from an ant gland depending on the size, food availability, breed of the ant, and expertise to collect the venom. In the present study, 35 ants were subjected to the extraction of the venom.

### Experimental design and ethics approval

Wister rats (males, 220-270 g, ≈ 20 weeks old) were obtained from Departmental Animal House (Zoology Department, King Saud University, Riyadh). They were housed in pathogen-free facilities maintained at 22 ± 2°C with 45-65% of relative humidity and 14/10-hour light/dark cycle. It is reported that the dose of SAV at 600 μg/kg affected physiologically and histopathologically treated rats [17]. Besides, the subcutaneous dose of the venom has been applied in several studies to induce colonic precancerous and cancerous model in rats [18]. The venom has been extensively studied to check its wound healing properties in open wounds on rat skin [19]. In the present investigation, the rats were assigned into three groups (n = 10) - control (injected with 1 mL of saline only), intraperitoneal (IP) injection of SAV, and subcutaneous (SC) injection of SAV. The five doses of SAV (600 μg/kg in 1 mL to each rat) were administered on every third day in both IP and SC groups. After 24 hours of the last injection, all rats were sacrificed and the samples collected for analysis. All the experiments conducted on animals were approved by the Departmental Animal Ethical Committee (College of Science, King Saud University, Riyadh) under approval number 3/2/110623.

### **Toxicity test of SAV *in vivo***

The acute toxicity of the crude SAV was assessed by using Wister rats. Sixty rats, after adapting for three days, were randomly divided into 12 groups (n = 5). The pure extract of SAV was dissolved in deionized water. All rats were allowed free access to fresh water and food after administration. Bliss assay was used to calculate LD_50_ by recording cumulative mortality within 14 days [20].

### Assay of biochemical parameters

*Serum biochemistry*

The blood from the retro-orbital venous sinus of each rat was collected into two tubes, one with EDTA for FACS analysis, and the second for serum. Blood samples were centrifuged at 3000 rpm for 10 minutes at 4ºC. The sera were separated and stored at -25ºC. The serum concentrations of both cholesterol and triglyceride were evaluated using kits (Quimica Clinica Aplicada S.A., Spain) for spectrophotometry (Pharmacia Biotech, UK). 

*Estimation of lipid peroxidation (MDA) and reduced glutathione (GSH)*

About half of the spleen from rats of each group was used for the assessment of lipid peroxidation (MDA) and glutathione (GSH). For this, the splenic tissues were homogenized (Automated homogenizer, IKA, T25D, Germany) in 10 mM KCl in 1.15% PBS and ethylenediaminetetraacetic acid (EDTA; pH 7.4) and centrifuged at 5000 × g for 10 minutes. The resulting supernatant was used to estimate the level of MDA and GSH by the established methods [21,22,23].

### Measurement of immune factors

The concentrations of major immune factors including IFN-γ and IL-17 were estimated in the sera by ELISA according to the manufacturer’s instructions for the corresponding rat immunoassay kits (Abcam, UK). The optical densities of the ELISA plate were measured at 450 nm. The level of sensitivity of the kits was 100 pg/mL.

### Fluorescence-activated cell sorter (FACS) analysis

For FACS of blood, the samples were collected in the non-EDTA coated tubes that were mixed 100 µL:2 mL with FACS lysing solution (Becton Dichinson, Germany). Dyes like fluorescein isothiocyanate (FITC)-anti-CD80 and phycoerythrin (PE)-labeled-anti-CD86, and MHC-DP+DQ+DR:PE monoclonal antibodies (Coulter Immunotech, France) were obtained to label the cells for analyses. After staining of the cells with antibodies (in dark at room temperature for 30 min), they were washed followed by their study in a FACS Calibur system using Cell-Quest software (Becton Dickinson, USA). A minimum of three events/sample was acquired. All results were expressed as the percentage of marker-positive cells in a respective gate.

### Histological studies

At necropsy, the liver and spleen of each rat were removed, weighed, and placed in neutral buffer in 10% formalin for 24 hours. The tissues were then processed in paraffin wax and were cut into 4-µm sections. After that sections were stained with haematoxylin and eosin (H&E) to highlight microscopic details of general histological architecture.

### Statistical analysis

The results were expressed as mean (M) ± standard deviation (SD). The one-way ANOVA statistical was analyzed by using MINITAB software (State College, PA, Version 13.1, 2002).

## Results

Two injection routes were employed in this study, the subcutaneous (SC) and the intraperitoneal (IP) injection of SAV. Results are summarized in Table 1.

**Table 1:** Different categories of test parameters for intraperitoneal and subcutaneous routes of SAV injection in male rats.

Categories of test parameter	IP route	SC route
Triglycerides (TAGs)	n	+
Cholesterol	n	+
Lipid peroxidation (MDA)	n	n
Reduced glutathione (GSH)	n	-
Interferon gamma (IFN-γ)	-	n
Interleukin-17 (IL-17)	-	n
MHC-II expression	+++	++
CD 80 expression	++	++++
CD 86 expression	++	++++
Spleen damage	++	+++
Liver damage	++	++++

n: no significant changes; +: increase; ++: twofold increase; +++: three-fold increase: ++++: four-fold increase; -: decrease; IP:intraperitoneal route; SC: subcutaneous route. All the fold-increase and decrease were compared with the control under histological analysis.

### The effect of SAV on the lipidogram

**Figure 1:**
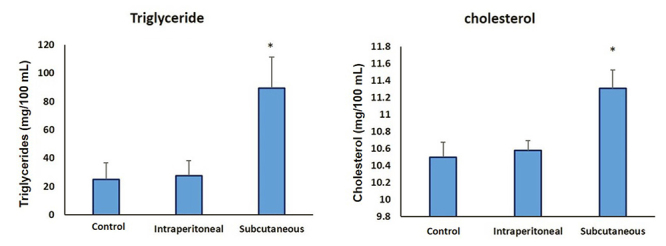
Levels of cholesterol and triglycerides in different rat groups, namely, control, intraperitoneal SAV-injected and subcutaneous SAV-injected rats.

The rats treated with subcutaneous injection of SAV showed significant increase in the concentrations of both total cholesterol and triglycerides by 7.6% and 260%, respectively, compared to the control goroup (Figure 1). In contrast, the intraperitoneal injection of SAV did not alter levels of these two parameters concerning the control to that extent.

### The effect on the lipid peroxidation and glutathione

**Figure 2:**
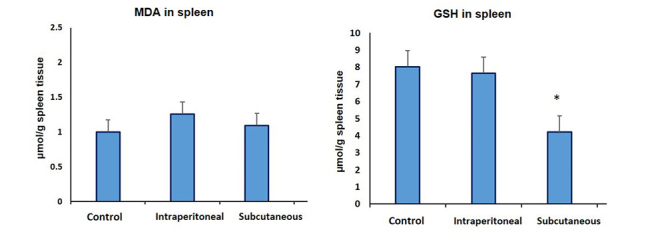
Levels of lipid peroxidation and glutathione in different rat groups, namely, control, intraperitoneal SAV-injected and subcutaneous SAV-injected rats.

The level of MDA and GSH was not significantly changed in rats treated via IP route as compared to the control group (Figure 2). However, rats that had received SAV via the SC route evidenced a decrease in total glutathione concentration by ~50% when compared to controls (Figure 2).

### Estimation of IFN-γ and IL-17

**Figure 3:**
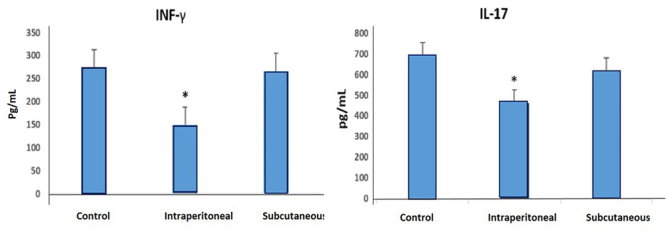
Levels of IFN-γ and IL-17 in different rat groups, namely, control, intraperitoneal SAV-injected and subcutaneous SAV-injected rats.

IP injection of SAV resulted in a significant decrease in IFN-γ and IL-17 levels by 40.01% and 35.7%, respectively, when compared with the control group (Figure 3). In comparison, no significant change was observed in the level of both cytokines in rats injected via SC route.

### PMNs, MHC-II, CD80, and CD86 expression

**Figure 4:**
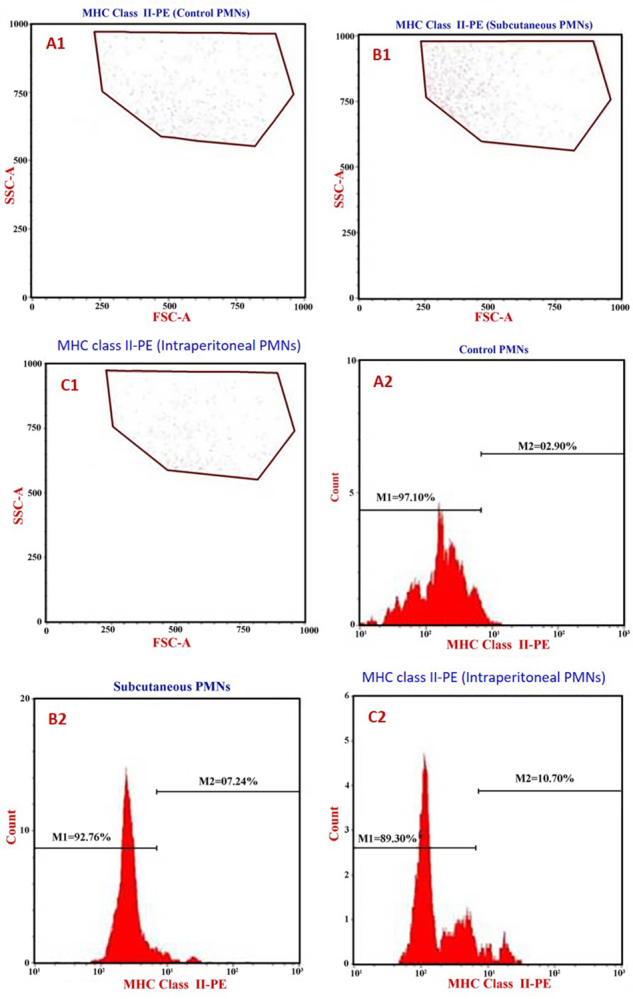
Representative cytoflourometry graphs of the MHC class II induction in the whole blood PMNs. **(A1, A2)** Unstimulated PMNs (control). **(B1, B2)** Subcutaneous SAV-stimulated PMNs. **(C1, C2)** Intraperitoneal SAV-stimulated PMNs.

FACS analysis of the samples showed that MHC-II expression on PMNs of control rats was 2.9% (Figure 4A). This value was significantly lower than that of rats that had received the SC SAV injection (7.2%, Figure 4B) or the intraperitoneal (IP) injection (10.7%, Figure 4C).

**Figure 5:**
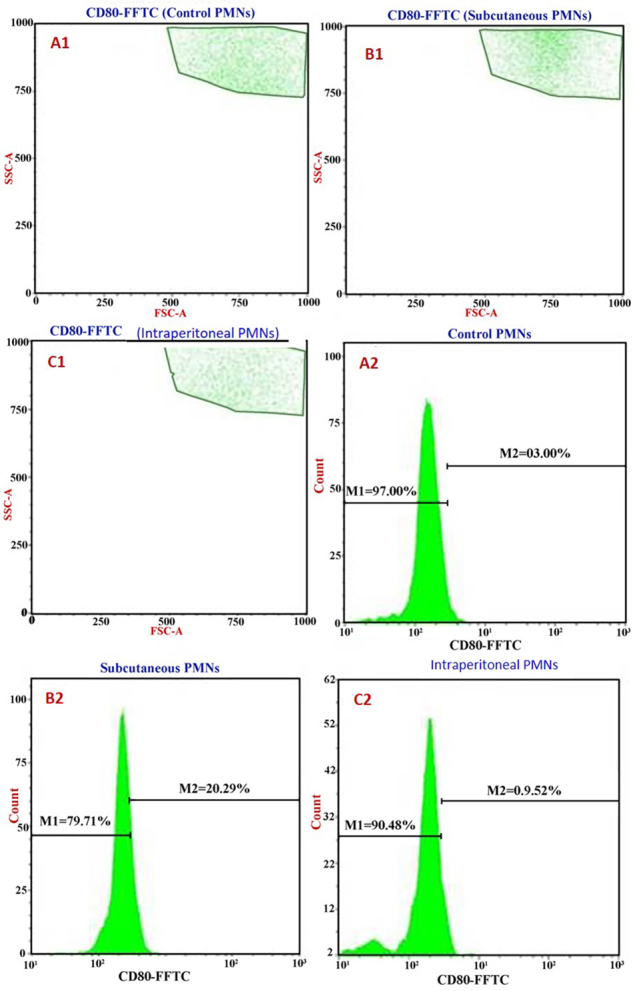
Representative cytoflourometry graphs of the CD80 induction in the whole blood PMNs. **(A1, A2)** Unstimulated PMNs (control). **(B1, B2)** Subcutaneous SAV-stimulated PMNs. **(C1, C2)** Intraperitoneal SAV-stimulated PMNs.

**Figure 6:**
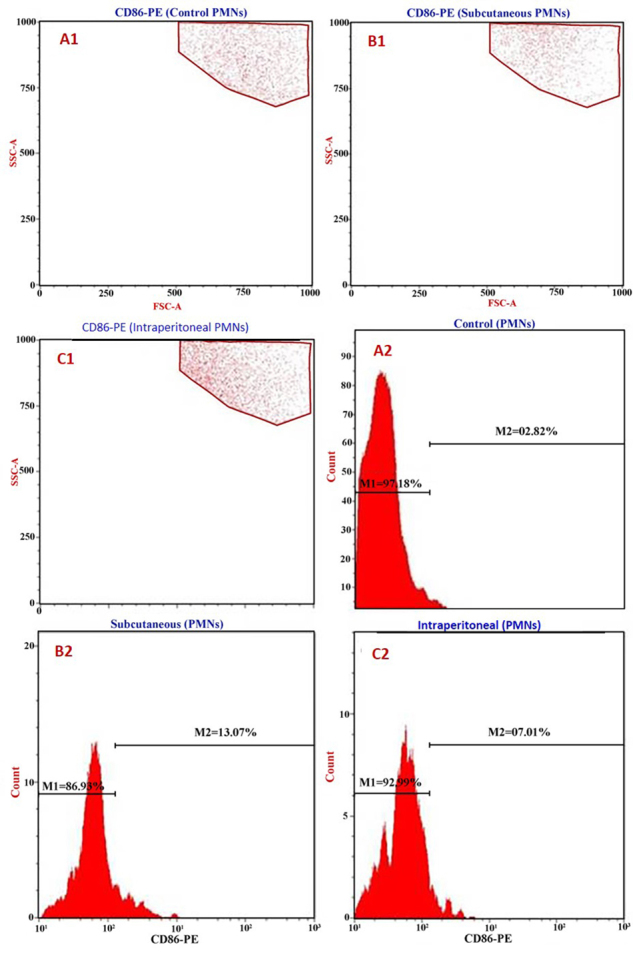
Representative cytoflourometry graphs of the CD86 induction in the whole blood PMNs. **(A1, A2)** Unstimulated PMNs (control). **(B1, B2)** Subcutaneous SAV-stimulated PMNs. **(C1, C2)** Intraperitoneal SAV-stimulated PMNs.

Analyses of CD80 expression on host PMNs showed that control rat cells had a level of 3.0% (Figure 5A). Again, this value was significantly lower than that observed in cells from rats that had received the SC SAV injection (20.3%, Figure 5B) or the IP injection (9.5%, Figure 5C). These same patterns were also evident with regard to analysis of PMN CD86 expression, i.e., while control rat PMN had 3.0% expression levels (Figure 6A), there were significant differences between controls (3.00%), subcutaneous-injected (13.07%) and intraperitoneally-injected (7.01%) hosts (Figure 6).

### Histopathological alterations

*Spleen*

**Figure 7:**
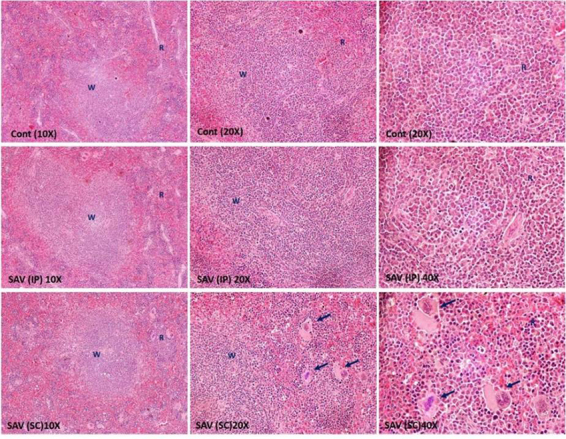
Representative photomicrographs of spleen tissue showing white (W) and red (R) pulps. Arrows show the megakaryocyte infiltration. Each group has three magnifications (10×, 20×, 40× and HE). Many rat spleens were histologically examined for pathological changes. Cont: control group, SAV: samsum ant venom, IP: intraperitoneal, SC: subcutaneous.

Histologic examination of the spleens indicated there were effects on the white and red pulp of rats that received SAV via IP route (Figure 7). Depletion of lymphocytes and infiltration of megakaryocytes were also noted in these rats. The histological examination of the splenic tissues in Figure 7 revealed severe and intense alterations in SC SAV-treated animals. A depletion of the white pulp lymphocytes was remarkably noted compared to the healthy tissues. Infiltration with megakaryocytes and different other cells was clearly observed in tissue of SC SAV-treated animals.

*Liver*

**Figure 8:**
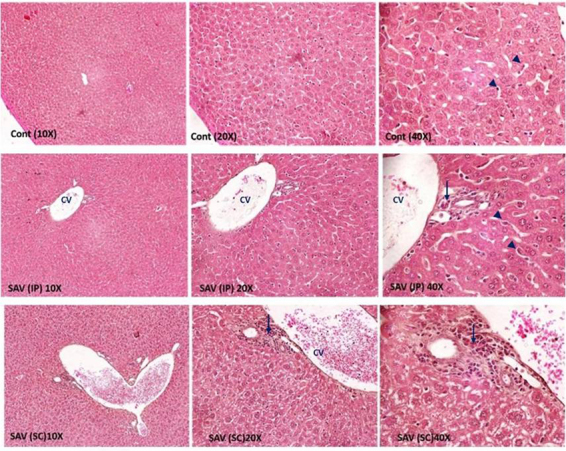
Representative photomicrographs of liver tissue. Each group has three magnifications (10×, 20×, 40× and HE). Arrows indicate inflammatory cells whereas arrow heads show Kuppffer’s cells. CV: central vein, Cont: control group, SAV: samsum ant venom, IP: intraperitoneal, SC: subcutaneous.

Examination of the liver tissue showed that SAV was able to induce alterations. IP-SAV injected rats revealed mild changes. Infiltration of the hepatic tissue with inflammatory cells was observed in IP SAV-treated rat group with dilatation in the central vein with enlargement of the kupffer cells (Figure 8). On the contrary, severe histopathological alterations were observed in SC SAV-treated animals. Cytoplasmic vacoulations were observed into hepatocytes. The central vein in the animals was dilated and most of their sinusoids were remarkably narrow in shape. Severe infiltration of different inflammatory cell types was observed in the examined sections from SC SAV-treated animals. Hence, histopathological examination revealed that SC SAV-treatment exerts the more toxic effect of SAV than IP treatment on tested animals (Figure 8).

## Discussion

Most ants inject secretions containing a range of bioactive elements in their bites that elicit inflammatory effects characterized by an increase in vascular permeability and neutrophil migration [24, 25, 26]. Although these effects have been thoroughly studied, the mechanism involved is poorly characterized up to this moment. We documented earlier how the low dose (100 μg/kg dose of SAV) against lipopolysaccharides (LPS) influences AKT1, Fas, TNF-α and IFN-γ mRNA expression in rats [18]. Herein, we have induced some immunological modulations by triggering the immune response with a high dose of SAV (600 μg/kg of body weight) in rat model.

In the present study, SC SAV treated rats exhibited a profound elevation in total cholesterol and triglyceride levels. Recently, many studies using animal models in high fat and cholesterol diet showed a remarkable increase in circulating levels of lipocalin 2 and its hepatic expression. These modifications were associated with increased infiltration of neutrophils [27]. Therefore, neutrophils seem to get activated and then migrate towards high-fat concentration sites *in vivo*. The coronary heart disease-bearing patients express PMNs, which have lower phagocytosis and ROS-mediated destruction of the tissue/pathogens. Increasing age and high levels of cholesterol were found to be positively correlated in humans [28]. It is also important to mention that an improved lipid profile promotes efficient angiogenic stimulus and re-epithelialization during the wound healing process [29].

Numerous studies in the literature suggest that serum lipids, including cholesterol and TAGs, play a significant role in executing an appropriate immune response while encountering an infection or in autoimmune diseases [30, 31]. It is also reported that unsaturated fats (EPA, DHA, and oleic acids) modulate the immune response [32]. Such unsaturated fats may decrease or increase the level of various cytokines depending on the concurrent internal and external environment of the biological system [33, 34, 35]. Lipids provide energy and signal modulations for cellular expansion and membrane remodeling which are essential for both innate and adaptive immune responses [36, 37]. In the present study, the level of cholesterol and TAGs increases moderately in comparison with the control post SAV treatment. This might be attributable to the elevation of immune response *in vivo*, including the expansion of immune and allied cells, modulation of their membrane fluidity and production of cytokines. 

Hence, it is also noteworthy that lipids regulate not only macrophage and T lymphocyte function but also their overall phenotype. The pathways that enhance lipid synthesis and accumulation lead to trigger a proinflammatory phenotype (Figure 2) while the other channels facilitating β-oxidation and lipid efflux dictate the immune cells towards an anti-inflammatory phenotype. However, both phenotypes are the result of an intricate network of various pathways, and the final cellular fate occurs in a context of tissue type and disease state [36].

We also observed that subcutaneously injected SAV caused a profound increase in expression of MHC-II together with the co-stimulatory molecules CD80 and CD86 (for migration) concomitant with a high concentration of both triglycerides and cholesterol in rats. Moreover, histological analysis showing hepatic infiltration with inflammatory cells in the same treated rats confirms the hypothesis. 

The splenic homogenate exhibited no significant change in the level of MDA and GSH in rats injected with SAV through IP route. However, IP injection of SAV led to a significant decline in the concentrations of both cytokines - IFN-γ and IL-17 - in comparison with the control group. Studies based on murine models suggest that infection raises the levels of IFN-γ and PMN in lung and spleen tissue [37]. Besides, another immune parameter, NF-κB, is stimulated by oxidative stress, which is a central regulator of inflammatory and immune responses. Hence, its regulation ceases the level of crucial pro-inflammatory cytokines, such as IL-1β, TNF-α, IFN-γ and IL-6 [38]. 

IFN-γ is the principal cytokine produced during TH1-type immune responses that are also released in response to IL-12. These results entail that SAV suppressed T-cell immune response. Many researchers have shown that isolated PMNs released IFN-γ after IL-12 and TNF-α stimulation *in vitro* while other investigators failed to detect IFN-γ after LPS stimulation [39]. Herein, it was found that isolated PMNs did not release IFN-γ after SAV stimulation *in vitro*. The pro-inflammatory cytokine, IFN-γ, promotes Th1 responses, which down-regulate the Th2-like immune responses that are hallmarks of allergic diseases. Hence, the allergy associated with the SAV on humans may be due to the decrease in the circulatory IFN-γ in the present study. 

Although activated CD4^+^ T-cells are believed to be a major source of IL-17, activated CD8+ T-cells, PMNs and eosinophils also produce IL-17 [40,41]. IL-17 is a pro-inflammatory cytokine that acts synergistically with TNFα and IL-1 [42]. It was found that IL-17 production by cultured splenocytes was not affected in mice receiving anti-CD80 mAb [43]. Similarly, here, the IP injection of SAV was found to decline the level of IL-17 in blood samples with a significant upregulation of CD80 and CD86. However, it has been revealed that the enhancement of PMN infiltration and macrophage function was associated with markedly increased IL-17 in serum [4]. 

**Figure 9:**
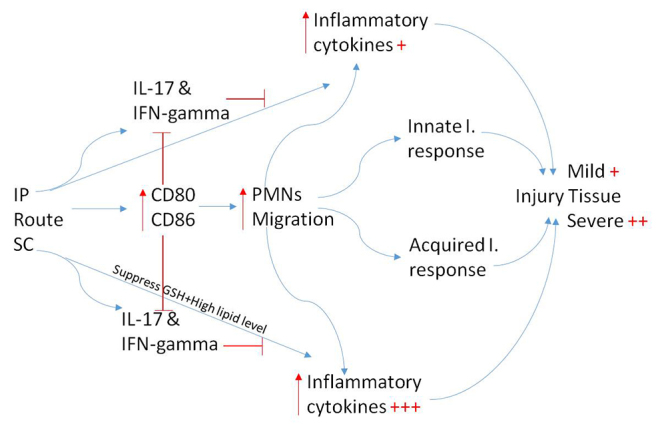
A summary of the effect of the two injection routes, intraperitoneal (IP) and subcutaneous (SC). Both IP and SC injections upregulate the expression of CD80 and CD86 on the PMNs (red arrows), and this directly support migration. Inflammatory cells increase cytokine secretion. By supressing GSH and elevating lipogram, SC was found to enhance tissue damge, (++) and this may be due to increase inflammatory cytokines (+++). Results showed that upregulation of the expression of CD80 and CD86 did not affect IL-17 and IFN-γ in SC rats (blocked line) and it was associated with a remarkably decrease of these two cytokins in IP rats.

In another study, the blockade of CD80 and CD86 reduced IL-17 production. Although the severity of some diseases such as joint inflammation can be affected by various cytokines including Th17-associated IL-17, our results suggest that another pathway - by which CD80 and CD86 may contribute to the disease pathogenesis and tissue damge - is not upregulated by IL-17. Here, CD80 and CD86 may contribute to hepatic and splenic tissue damge through enhancing various inflammatory cytokines such as TNFα and IL-1. In particular, SC route of SAV injection was more efficient than IP by disturbing oxidative stability (GSH decrease) and increasing lipogram concentration. This in turn may stimulate secretion of inflammatory cytokines that induce tissue damage (Figure 9). 

The histological analysis confirms the biochemical and immunological results, showing the depletion of lymphocytes in the white pulp in IP SAV treated rats. It suggests a reduction in the lymphocyte number in peripheral blood and lymphoid organs that might be attributable to the significant reduction of IFN-γ in plasma, which stimulates IL-2 and IL-7 secretion. The dramatically declined lymphocyte number may indicate that lymphocytes are stressed by SAV toxicity starting with high levels of free radicals, increasing the levels of pro-inflammatory cytokines and ending by programmed cell death. Thus, results demonstrated that SAV may be capable of inducing splenocytic apoptosis. 

The cytoplasmic vacuolation in hepatocytes is mainly a consequence of disturbance in lipid inclusions and metabolism during pathological changes. The vacuolar degeneration has been regarded by Ebaid et al. [44] to be an alteration produced to collect the injurious substances in the hepatocytes. The lymphocyte infiltration in the hepatic tissue is suggested to be a prominent response of body tissues facing injurious impacts. 

Recently, a study demonstrated an association of PMNs with a specific IL-17/IL-22 environment in HIV-infected patients with highly activated PMNs [45]. This might be the reason for upregulation of MHC-II and the costimulatory molecules CD80 and CD86 on the PMNs after injection of SAV intraperitoneally and subcutaneously in rats. The activation and recruitment of PMNs can be stimulated by IL-15, IFN-γ, CSF-CSF, and IL-8 [46,47]. Therefore, it is evident that the subcutaneous injection of SAV activates stronger stimulated PMNs than the intraperitoneal injection, which can be due to physiological and immunological alterations. This explains the significant lower expression of CD80 and CD86 on PMNs of the IP injection (9.5%, and 7.01%) in comparison with PMNs of the SC SAV injection (20.3%, and 13.07%) in the present work.

## Conclusion

The present study indicates that stimulation of PMNs by SAV leads to upregulation of MHC-II, CD 80, and CD 86. These molecules play a critical role in antigen presentation and, consequently, in proliferation of T-cells [48]. Hence, SAV is very effective in orchestrating the appropriate immune response by triggering acquired immune response indirectly. Moreover, the efficacy of SAV is more pronounced by subcutaneous administration in regard to intraperitoneal one. This aspect of SAV can be useful in the treatment of wide array of infections, autoimmune diseases and cancer. 

### Abbreviations

CD80 and CD86: costimulatory molecules; FACS: fluorescence-activated cell sorter; GSH: reduced glutathione; HLA-II: human leukocyte antigen class II; IFN-γ: interferon gamma; IL-17: interleukin-17; IP: intraperitoneal; LPS: lipopolysaccharides; MDA: lipid peroxidation; MHC-II: major histocompatability class II; PBS: phosphate buffered saline; PMNs: polymorphonuclear neutrophils; SAV: samsum ant venom; SC: subcutaneous; TAGs: triglycerides; TNF: tumour necrosis factor.
